# Notch signaling in leech neurogenesis: expression patterns and functional insights in the Glossiphoniid leech *Helobdella austinensis*

**DOI:** 10.1080/19768354.2026.2631845

**Published:** 2026-02-23

**Authors:** Geon-Hwi Jeong, In-Hyeok Pyo, Yam Prasad Aryal, Geon-Woo Lee, Kyoung-Bin Ryu, Hee-Jin Kwak, Sung-Jin Cho

**Affiliations:** a Department of Biological Sciences and Biotechnology, College of Natural Sciences, Chungbuk National University, Cheongju, Chungbuk, Republic of Korea; b Division of Developmental Biology, Cincinnati Children's Hospital Medical Center, Cincinnati, OH, United States; c Department of Biology Education, College of Education, Kongju National University, Gongju, Republic of Korea

**Keywords:** Notch signaling, neural development, DAPT, Helobdella austinensis, lophotrochozoans

## Abstract

The Notch signaling pathway is one of the most evolutionarily conserved pathways, playing a crucial role in juxtacrine signaling between adjacent cells. Although previous studies have characterized Notch signaling in diverse models, its function in the lophotrochozoan animals is largely not understood and is only partially identified in early-stage embryos. To address this gap, we reaffirmed the expression of Notch homolog, including downstream components, in the organogenesis of the Glossiphoniid leech, *Helobdella austinensis*. We analyze the spatial and temporal expression patterns of the Notch receptor (*Hau-notch1*), ligands (*Hau-delta* and *Hau-jagged*), and transcription factors (*Hau-hes* and *Hau-hey*) from embryonic stages 8–11. Notch transcripts are expressed in the germinal plate and segmental precursor cells at stage 8, with expression expanding to the somites, ventral and supraesophageal ganglia by stage 9. In organogenesis stages (stages 9–11), Notch components are expressed in the anterior and posterior suckers, proboscis, and ventral ganglia, indicating their role in nerve differentiation. Furthermore, blocking of Notch signaling by DAPT (N-[N-(3, 5-difluorophenacetyl)-l-alanyl]-s-phenylglycine t-butyl ester) leads to the disruption of nerve formation with downregulation of transcription factors (*Hau-hes* and *Hau-hey*). Our findings provide insights into the conserved mechanisms of Notch signaling in bilaterian neural development, contributing to a deeper understanding of evolutionary developmental biology.

## Introduction

As part of the development and maintenance of multicellular organisms, intercellular communications are essential. It has been shown that a variety of signaling pathways are involved in cell to cell communication during development, cell fate, symmetry, apoptosis, compartment boundary formation, somitogenesis and the formation of compartment boundaries (Jafar-Nejad et al. 2010; Cheon et al. 2024; Hong et al. 2024; Moon et al. 2025). As one of the most evolutionarily conserved pathways, Notch signaling is known for its role in juxtacrine signaling pathways between adjacent cells (Kopan and Ilagan 2009). There are a number of multicellular organisms that rely on Notch signaling to develop and maintain multiple tissues and organs. The activation of Notch signaling occurs when a ligand (Delta, Serrate or Jagged) binds to a receptor (Notch) of the neighboring cell, resulting in the activation of Notch signaling. After cleavage, the Notch receptor intracellular domain (NICD) is liberated from the membrane, which leads to subsequent cleavages. Following binding to Supressor of Hairless [su(H)], NICD enters the nucleus and binds to Su(H). However, upon binding to NICD, Su(H) serves as a transcriptional activator together with other cofactors (Yuan et al. 2016). There are several conserved Notch target genes, including hairy and Enhancer of split-related genes belonging to the orange domain-containing bHLH transcriptional repressor family (bHLH-O genes). There is evidence that bHLH-O genes can target themselves, leading to auto-inhibitory feedback loops that cause their expression to oscillate dynamically (Davis and Turner 2001; Hirata et al. 2002; Gratton et al. 2003; Giudicelli and Lewis 2004; Zhou et al. 2012).

During the development of vertebrate embryos, the paraxial mesoderm segments into temporary structures called somites. These somites emerge sequentially as partially epithelialized cell clusters that originate at the anterior region of the presomitic mesoderm (PSM). Cooke and Zeeman (Cooke and Zeeman 1976) initially proposed a clock-and-wavefront model to explain this segmentation process, but extensive molecular evidence (Lewis 2003) has since supported it. Based on the discovery that hairy, a transcriptional repressor targeted by Notch, oscillates in the chick PSM, the concept of a ‘segmentation clock’ was molecularly supported (Palmeirim et al. 1997). There is a common feature of segmentation in the body plans of diverse animal groups, including annelids, arthropods, and vertebrates. Nevertheless, it is not clear whether these types of segmentation developed independently or share a common evolutionary origin (Graham et al. 2014). Despite controversy regarding Notch signaling, past and recent studies suggest that Notch signaling is important for the development and regeneration of neurons in invertebrates (Lv et al. 2024).

Compared to oligochaetes and polychaetes, leeches lack segmental bristles (chaetae) and possess characteristic organs called suckers in their anterior and posterior ends. There are 32 true segments within a leech. Rostral end of the animal comprises four segments, designated R1-R4, which are fused to compose head brain; midbody segments M1–M21, each with each own segmental ganglion; caudal segments C1-C7, which compose the fused tail brain (Weisblat et al. 1988; Weisblat and Kuo 2014) In Helobdella, segmentation is driven by a posterior growth zone (PGZ) comprised of five bilateral pairs of individually identified segmentation stem cells (teloblasts). *Helobdella robusta* has previously been studied for the expression and function of several Notch pathway components (Gonsalves and Weisblat 2007; Rivera and Weisblat 2009). The leech homolog of the hairy and enhancer of split (HES) genes, *Hro-hes*, was strongly expressed during segmentation (Song et al. 2004; Rivera et al. 2005), as well as the leech homolog of the Notch receptor, *Hro-notch*. However, there is still a lack whether Notch signaling and its components contributes neurodevelopment in the organogenesis stages.

Recently, the whole genome of *H. austinensis* was sequenced (Schultz et al. 2024a), which allows to retrieve complete components of the signaling pathway. The accessible genome enables us to conduct a deeper investigation of the Notch signaling pathway and its involvement in leech embryogenesis. In this study, we identified Notch signaling components, including homologs of the receptor Notch (*Hau-notch1 and Hau-notch2*) and the ligand Jagged and Delta (*Hau-jagged* and *Hau-delta*), as well as more potential bHLH Notch target genes, including one Hey family member, *Hau-hey*, and another hairy and Enhancer of split-related gene, *Hau-hes*. Herein, we identified the spatial expression pattern of components to examine the evidence of neurodevelopment in *H. austinensis*.

Furthermore, to demonstrate a comprehensive role of Notch signaling in the *H. austinensis* embryogenesis, we performed the drug treatment related to the inhibition of Notch signaling. DAPT (N-[N-(3,5-difluorophenacetyl)-l-alanyl]-s-phenylglycine t-butyl ester) is one of the well-known inhibitors of γ-secretase, which prevents cleavage of Notch intracellular domain (NICD) and consequently blocks the Notch pathway (Dorneburg et al. 2016).

## Material & method

### Animal

*H. austinensis* were raised in the Laboratory of Regeneration and Evolutionary Development (Chungbuk National University, Republic of Korea). We got leech embryos by following previously established protocols (Weisblat and Kuo 2009; Gline et al. 2011; Kutschera et al. 2013). To remove debris, the leech bowls were wiped once a day, and fed every other day. They were stored at room temperature (22°C).

### Alignment and phylogenetic analyses

We identified Notch components in the genome data of *H. austinensis*. Using homologs from representatives of the major metazoan clades, they were constructed based on their complete amino acid sequence. Alignments were carried out with the MUSCLE (Multiple Sequence Comparison by Log – Expectation) tool in MEGA X software. MEGA X software was used to analyze the aligned sequences (Kumar et al. 2018) using the model selected based on finding the best-fit model (Santos et al. 2020). The Maximum Likelihood analyses were performed by generating 1,000 bootstrap replicates. In the phylogenetic analysis of Notch, only a part of the sequence from the LNR domain to the end was contained in the alignment. As the number of EGF domains present in Notch proteins is variable, they were not considered in the phylogenetic tree (Gazave et al. 2009).

### Gene identification and cloning

In *H. austinensis* whole genome data, we identified genes: *Hau-notch1*, *Hau-notch2* as receptors, *Hau-delta* & *Hau-jagged* as ligands and *Hau-hes, Hau-hey* as transcription factors. Thereafter, primers of each gene were designed (Table S1). RNA extraction and cDNA synthesis were performed according to the manufacturer's instructions as described previously (Kwak et al. 2022; Pyo et al. 2025). Following the manufacturer's instructions, TaKaRa Ex Taq® was used to perform PCR under the following cycling conditions: predenaturation at 94°C for 5 min, denaturation at 94°C for 30 s, variable annealing temperature for 30 s, variable extension time at 72°C, and post-extension at 72°C for 5 min. In the next step, the amplified fragments were cloned into the pGEM T vector (Promega, Madison, WI, USA).

### Probe synthesis for *in situ* hybridization

RNA probe was constructed for the expression analysis of Notch components. Cloning of the components was carried out with pGEM T vector (Promega, Madison, WI, USA). RNA probes were made using the MEGAscript kit (Ambion, Austin, TX, USA) and DIG or Fluorescein RNA Labeling Mix (Roche, Basel, Switzerland), which referred to the manufacturer's protocol. The synthesized RNA probes were applied to each sample at a final concentration of 4 ng/μl.

### Fluorescence *in situ* hybridization

To profile the expression patterns of Notch components in *H. austinensis,* double fluorescence whole-mount *in situ* hybridization was carried out using the NEN (New England Nuclear) tyramide signal amplification (TSA) Plus kit (PerkinElmer, Wellesley, MA, USA). We followed the protocol as described by Kwak et al. 2022; embryos were fixed in absolute MeOH for dehydration. Before treated RNA probe in the embryos, we treated 20 μg/mL Proteinase K (Biofact, Daejeon, South Korea), glycin mixture (2 mg/ml) and 1M triethanolamine (TEA, pH 8.0; Sigma-Aldrich) with acetic anhydride for the permeation of the probe. Next, we treated the 4% PFA for post-fixation. Hybridization was performed using 4 ng/μl riboprobe in a hybridization buffer (50% formamide, 5× saline-sodium citrate [SSC], 1× Denhardt's solution, 0.1% CHAPS [3-[(3-cholamidopropyl) dimethylamino]−1propanesulfonate], 100 µg/mL heparin, 0.1% Tween 20, and 100 µg/mL transfer RNA) at 64.7°C for 20 h. After the hybridization step, we washed the samples with SSC and PBT, then anti-DIG/POD (In chemical, using anti-DIG/AP) (1:1000) with western blocking solution (1:9 10× Roche Western Blocking Reagent in PBT) at 4°C for 16 h. After blocking, TNT buffer (0.1 M Tris-HCl, pH 7.5; 0.15 M NaCl; 0.1% Tween 20) was used for the washing. We treated color reaction through the addition of reconstituted TSA PLUS Cyanine-3 Reagent, diluted 1:50 in NEN Amplification Solution for 7 min. For chemical, washing was carried out with PBT and color reaction was carried out using NBT/BCIP (Roche, Basel, Switzerland), diluted 1:50 in PBT. For double *in situ* hybridization, two different types of RNA Labeling Mix (DIG RNA Labeling Mix or Fluorescein RNA Labeling Mix), each targeting different genes were treated at the same time. After these steps, the color reaction was performed as previously described. After that, blocked with anti-Fluorescein/POD (1:1000) at 4°C for 16 h and the procedure with the color reaction initiated through the addition of reconstituted TSA Plus Fluorescein Reagent, diluted 1:50 in NEN Amplification Solution for 10 min once again. Finally, after these steps, samples were visualized using Zeiss AXIO Zoom.V16 (Carl Zeiss, Oberkochen, BW, Germany) and confocal microscope (Leica, Wetzlar, HE, Germany).

### Immunofluorescence

Immunofluorescence was carried out using stage 10 embryos. The embryos were treated in western blocking solution (1:9 10× Roche Western Blocking Reagent in PBT) for an hour after rehydration. Then, monoclonal anti-acetylated-α-Tubulin antibody (Sigma, T-7451) in western blocking solution (1:800) was treated at 4°C for 20 h. After three consecutive washes with PBT, embryos were incubated with a secondary antibody (Alexa 488, Abcam, ab150113) in western blocking solution (1:250) at 4°C for 20 h. After that, DAPI (in PBT, 1:1000) staining was carried out at room temperature in the dark for 7 min. After washing with PBT 3 more times, embryos were embedded in 30%, 50%, 20 min, and 87% glycerol and 2.5 mg/ml of 1,4-Diazabicyclo[2.2.2]octane (DABCO; Alfa Aesar, USA) in 1xPBS. Embryos were imaged by fluorescence microscopy on Zeiss AXIO Zoom.V16 and Leica DM6 B.

### Drug treatment and RT–PCR

At stage 7, *H. austinensis* embryos were incubated in Helobdella triserialis (HTR) saline medium (4.8 mM NaCl, 1.2 mM KCl, 2 mM MgCl2, 8 mM CaCl2, and 1 mM maleic acid), including drugs: DAPT (SigmaAldrich, Saint Louis, MO, USA), a Notch signaling antagonist. Total DAPT concentration was 10 μM in HTR media for 24 h.

Total RNA was isolated from DAPT-treated and control *H. austinensis* embryos at stage 10 using TRIzol (Invitrogen, Carlsbad, CA, USA). mRNA was purified from Total RNA with Oligo (dT) primer (Promega, Madison, WI, USA), and reverse transcribed into cDNA with a SuperScript II First-Strand Synthesis System for RT–PCR (Invitrogen, Carlsbad, CA, USA). After synthesis, the system was normalized using *GAPDH* (forward primer: 5’-GAACGAGGATGGCTACAAGAA-3’, reverse primer: 5’-GTGTACGAGTGGATGGTGG-3’) (Kwak et al. 2019). The qPCR mixture included a 0.5 μl of forward primer, and 0.5 μl of reverse primer (Table S1) along with 10 μl 2X qPCR buffer, 4 μl SYBR, 1 μl selected cDNA and 2 μl ROX in Applied Biosystems™ PowerUp SYBR Green Master Mix for qPCR with 10 μl total reaction volume per well using the StepOnePlus Real-Time PCR system running the following program: step 1: 95°C for 3 min for an initial melting, 95°C fir 15 s, 60°C for 1 min for 40 cycles, followed by a melt curve analysis. Quantitative values were calculated using StepOne Software v2.3 (Applied Biosystems, Foster City, CA, USA).

## Results

### Identification and selection of Notch signaling pathway genes

In this study, in order to analyze the function of the Notch signaling pathway in *H. austinensis*, genes representing the key components of the pathway were selected as analysis targets in *H. austinensis* WGS data. Delta & Jagged and Notch were selected as ligands and a receptor, and Hes and Hey genes were selected as downstream transcriptional factors. These genes were verified that the highly functionally important domains preserved. In particular, Notch, Delta, and Jagged are key components responsible for signaling, and Hes and Hey are known as representative downstream target genes of the pathway.

### Phylogenetic analyses and domain composition of leech Notch components

**Figure 1.**
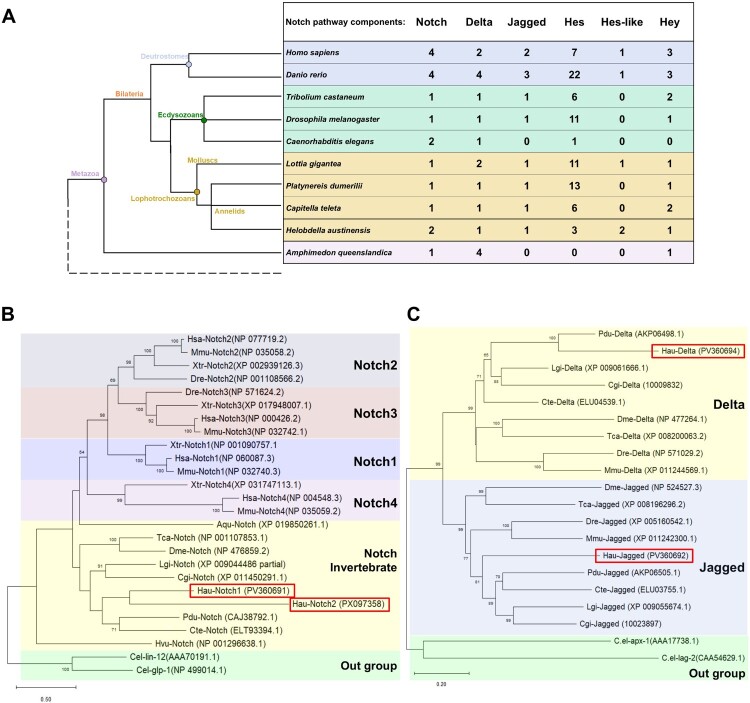
(A) The number of gene copies of Notch main components in metazoans. Ten species representatives of the metazoans, included two deuterostomes: three ecdysozoans, four lophotrochozoa and one out group (porifera). (B-C) Phylogenetic analysis of Notch core components in *H. austinensis*. (B) Phylogenetic tree for Notch class genes. (C) Phylogenetic tree for Delta and Jagged class genes. Species abbreviations: Aqu, *Amphimedon queenslandica*; Cgi, *Crassostrea gigas*; Cel, *Caenorhabditis elegans*; Cte, *Capitella teleta*; Dme, *Drosophila melanogaster*; Dre, *Danio rerio*; Hau, *H. austinensis*; Hsa, *Homo sapiens*; Hvu, *Hydra vulgaris*; Lgi, *Lottia gigantea*; Mmu, *Mus musculus*; Pdu, *Platynereis dumerilii*; Tca, *Tribolium castaneum*; Xtr, *Xenopus tropicalis*.

To identify the Notch gene family in *H. austinensis*, we retrieved core components of the Notch pathway. In *H. austinensis,* two *Notch* genes, one *Delta, Jagged, Hey* gene and three *Hes* genes were identified through whole genome sequence analysis (Schultz et al. 2024b). In this study, we focused on one Notch receptor (*Hau-notch1*), two ligands Delta (*Hau-delta*) and Jagged (*Hau-jagged*), and two transcription factor genes (*Hau-hes and Hau-hey*) of the underlying helix–loop–helix (bHLH) family, with a complete sequence and well-preserved domains (Figure 1A). From domain analysis of deduced amino acids, *Hau-Notch1* and *Hau-Notch2* present 35 Epidermal Growth Factor – like (EGF) repeats, three Lin-12/Notch Repeats (LNR), one Nucleotide-binding Oligomerization Domain (NOD), one Notch-specific Oligomerization/Processing Domain (NOPD) and six Ankyrin (ANK) repeats. In *Hau-Delta* and *Hau-Jagged*, those showed a Mastermind-like N-terminal (MNLL) domain, a Delta/Serrate/Lag (DSL) domain and a series of EGF repeats (seven for *Hau-Delta* and 15 for *Hau-Jagged*). In addition to these domains, *Hau-Jagged* also contains a Von Willebrand factor C domain (VWC) characteristic of Serrate/Jagged proteins and the JAG1 intracellular domain. For *Hau-Hes* and *Hau-Hey,* they contain the bHLH domain and the orange domain, and each of them has the characteristic domain WRPW and YRPW, respectively (Figure S1) (Chen et al. 2012). Phylogenetic analyses were performed to assess homology between Notch family genes across diverse metazoan species (Figure 1B, 1C, S2). Phylogenetic analysis of *Hau-notch1* was conducted alongside metazoan Notch receptors, focusing on the conservation of key structural domains such as Lin-12/Notch repeats (LNRs), and the negative regulatory region (NRR) (Figure 1B). Additionally, according to the shared presence of the MNLL and Delta/Serrate/Lag (DSL) domains, the phylogenetic relationships of *Hau-delta* and *Hau-jagged* were analyzed together (Figure 1C). Likewise, the transcription factor genes *Hau-hes* and *Hau-hey* were analyzed using the same approach (Figure S2A, S2B). Our results showed conserved Notch pathway components across metazoans.

Our study analyzed the temporal and spatial expression of both duplicate *Notch* genes (*Hau-notch1* & *Hau-notch2*) by ISH (*In situ* hybridization), and we found that there were no significant differences between the expression of *Hau-notch1* & *Hau-notch2*. For brevity, only the results for *Hau-notch1* are presented in the result part, but the following description applies to both *notch* paralogs.

### Anterior cell cluster-specific gene expression of Notch components in epiboly stage of *H. austinensis* embryo

**Figure 2.**
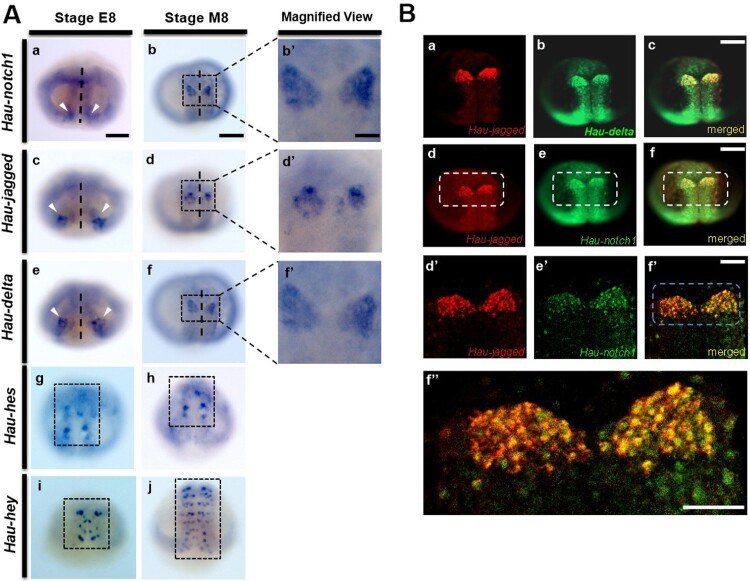
Notch main components (*Hau-notch1, Hau-delta* and *Hau-jagged*) are observed similar expression patterns during embryonic stage 8. **A** At early stage 8, they are broadly expressed in the segmental precursors (white arrowhead) at low levels with stronger expression in anterior clusters of cells (a, c and e). At mid-stage 8, the expressing cells gather at the anterior of the germinal plate (black square box) (b, d and f). The highly magnified view of Notch main components expression in stage 8 (b’, d’ and f’). *Hau-hes* is expressed in the segmental precursors at low levels with stronger expression in anterior clusters of cells (black box) during stage 8 (g-h). Unlike this, *Hau-hey* expression clusters of cells were located at the anterior of the left and right germinal bands (black square box) at the early stage 8. Additionally, pairs of cells expressed broadly on the germinal plate as they enter the germinal plate mid-stage 8 (i, j). **B** Expression of Notch main components (*Hau-notch1, Hau-delta* and *Hau-jagged*) in the anterior of germinal plate (a-f). In detailed images of confocal microscope (white box), the expression cells revealed significant spatial overlap signals in the anterior of germinal band (d’, e’, f’). High magnified image (blue box) of co-localizations of *Hau-jagged* and *Hau-notch1* (f’’). Scale bar: 100 μm (Aa, Ab, Bc, Bf and Bf’’); 25 μm (Ab’); 50 μm (Bf’).

To examine the developmental contribution of Notch components, we used stage 8 embryos due to arising of segmentation and neurogenesis by migration of neuronal cells (Weisblat and Kuo 2014; Kuo and Hsiao 2018). At early stage 8, the expression of *Hau-notch1, Hau-delta,* and *Hau-jagged* is observed in segmental precursor cells, with stronger expression in the anterior cell clusters (Figure 2Aa, Ac, Ae). By mid-stage 8, these genes are persisted along the germinal plate (Figure 2Ab, Ab’, Ad, Ad’, Af, Af’). Intriguingly, the two Notch downstream targets *Hau-hes* and *Hau-hey* exhibit distinct expression patterns during the coalition of germinal bands. At early stage 8, *Hau-hes* is expressed in a few cells localized in the coalesced germinal plate (Figure 2Ag-Ah), and *Hau-hey* is expressed in paired cells in each germinal bandlet (Figure 2Ai). At mid-stage 8, the expression of *Hau-hey* transcripts is reminiscent of ectodermal lineage-dependent genes such as *FoxA3* and *snail1* following the germinal plate process (Kim et al. 2017; Kuo and Hsiao 2018) (Figure 2Ai, Aj). Based on the spatial correlation between Notch ligands and their receptor, we performed double fluorescence whole-mount *in situ* hybridization at mid-stage 8 to identify spatial evidence of whether adjacent cells interact with each other in RNA expression level. Our results revealed that Notch ligand–receptor components are co-expressed in the anterior cell cluster of the germinal plate (Figure 2Ba-Bf). In addition, the highly magnified results also show that those ligands and receptors are expressed in the same cell along with its neighboring cells (Figure 2Bd’-Bf’’).

### Segmental-ganglionic and multifaceted expression of Notch components during organogenesis

**Figure 3.**
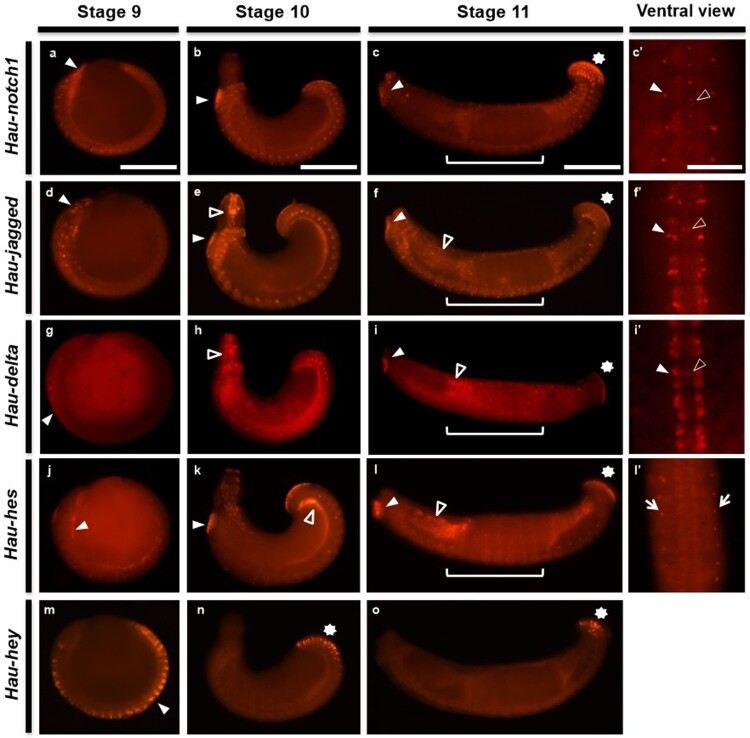
In early stage 9, *Hau-notch1, Hau-jagged and Hau-delta* were expressed in the dorsal ganglia of the head, which develop into ventral segmental ganglia and in micromere-derived supraesophageal ganglions in the prostomium (white arrowhead) (a, d, g). *Hau-hes* and *Hau-hey* were detected in the ventral segmental ganglia without expression patterns in the prostomium (white arrowhead) (j, m). In stage 10, the expressions of *Hau-notch1* were observed in the site of the anterior sucker and the dorsal midline of the ventral segmental ganglia (white arrowhead) (b). *Hau-jagged* and *Hau-delta* were observed throughout the developing everted proboscis (open arrowhead), as well as in the dorsal midline of the ventral segmental ganglia (white arrowhead) (e, h). *Hau-hes* was expressed in the visceral muscle (open arrowhead) and anterior sucker-like *Hau-notch* (white arrowhead) (k). On the other hand, *Hau-hey* was expressed only on the posterior sucker (white asterisk) (n). In stage 11, Notch components without *Hau-hey* were expressed on the anterior (white arrowhead), posterior sucker (white asterisk), proboscis (open arrowhead) and ventral segmental ganglia (white bracket), while *Hau-hey* was expressed only on the posterior sucker (white asterisk) (c, f, i, l, o). The ventral view of Notch components without *Hau-hey* (c’, f’, i’, l’). Notch core components (*Hau-notch1, Hau-jagged* and *Hau-delta*) were expressed on the segmental ganglion (white arrowhead) and dorsal midline (open arrowhead) (c’, f’, i’). *Hau-hes* was expressed on the peripheral neurons in the body wall (l’). Scale bar: 200 μm (a, b, c); 50 μm (c’).

After characterizing the expression patterns of Notch components at early stages, we next examined their expression in organogenesis embryos (stages 9–11, 155–310 h after zygotic development) to investigate the developmental expression during active organ differentiation. At stage 9, *Hau-notch1* is expressed in the prostomium and visceral precursor along with the ventral germinal plate (Figure 3a). During the proboscis eversion, the intense expression of prostomium is maintained in the anterior sucker precursor and *Hau-notch*1 transcripts are detected in visceral muscle, ventral ganglia with posterior sucker precursor (Figure 3b). At stage 11, referred to as proboscis invagination with gut differentiation stage (Kwak et al. 2022; Medina-Jiménez et al. 2025), *Hau-notch1* transcripts are detected in the mouthpart, anterior and posterior sucker, and gut boundaries (Figure 3c). Remarkably, *Hau-notch1* is expressed in likely neuropil glia following midline and root nerve cells of each segmental ganglion (Figure 3c’) (Tahtouh et al. 2009; Raffo-Romero et al. 2019; Kuo et al. 2024).

Similar to *Hau-notch1* expression, *Hau-jagged* is also expressed in prostomium and presumptive ectoderm lineage of germinal plate during stage9 (Figure 3d). After proboscis eversion, intense expression is detected in the proboscis cavity, visceral muscle, and precursors of the anterior and posterior sucker (Figure 3e) (Kwak et al. 2019). At stage 11, *Hau-jagged* shows a salt-and-pepper pattern in the whole body part with expression in suckers, gut boundaries, and segmental ganglia. In a magnified view of the ganglia region, *Hau-jagged* is expressed in neurons in lateral packets and presumptive Retzius (Rz) cell (Figure 3f’) (Heath-Heckman et al. 2021; Kuo et al. 2024). Another ligand *Hau-delta* showed only weak expression in the germinal plate (Figure 3 g). During proboscis eversion and invagination stages, the overall expression pattern is similar to *Hau-jagged* (Figure 3h). In stage 11, *Hau-delta* is expressed esophagus, gut boundaries and each sucker. Likewise to the ganglionic expression pattern of *Hau-jagged*, *Hau-delta* is also expressed in presumptive Rz cells and neuronal cells (Figure 3i’).

The downstream transcription factors exhibited distinct patterns; *Hau-hes* is expressed only in the visceral muscle at stage 9 (Figure 3j); however, salt-and-pepper pattern is shown in the developing proboscis, suckers, and visceral muscle, showing dot patterns in the lateral body from stage 10–11 (Figure 3k-3l). In a magnified view of the ventral region for detailed expression, those dot patterns are bilaterally expressed following segments likely in presumptive sensory cells related to sensilla (Figure 3l’) (Kuo and Lai 2019). Intriguingly, *Hau-hey* shows temporal expression following ectodermal tissue in the germinal plate. The expression is dimmed during organogenesis from the anterior to posterior end, and *Hau-hey* is limitedly expressed in the posterior sucker in stage 11 (Figure 3m-3o).

### Inhibition of Notch signaling induces nerve degeneration during gangliogenesis

**Figure 4.**
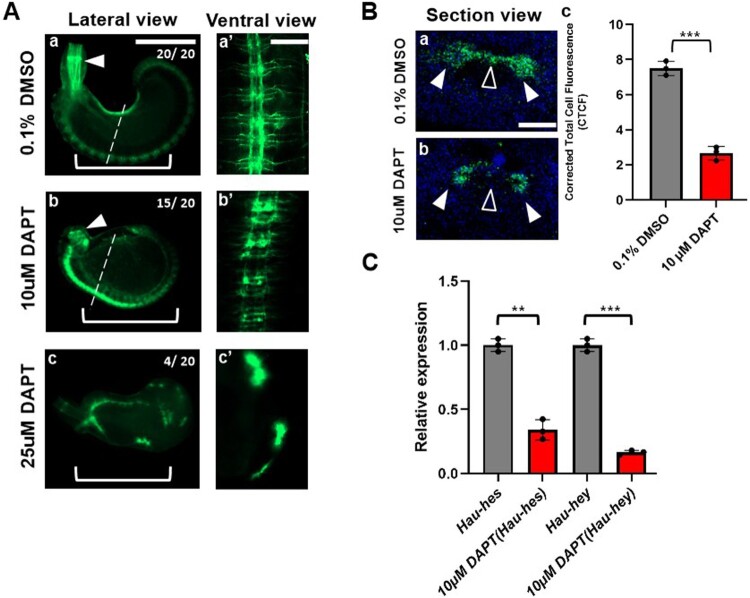
Inhibition of γ-secretase induces defects in the development of proboscis and VNC (ventral nerve cord) in *H. austinensis* with immunostaining for acetyl-α-tubulin (ac-tubulin). (A) Lateral and ventral views of stage 10 embryos are shown. Embryos were treated with DAPT 10, 25 µM, respectively, in DMSO. Immunostaining was expressed on the proboscis (white arrowhead), and VNC (white bracket). (B) Cross-section view of two individual stage 10 embryos (0.1% DMSO, 10 µM DAPT) for detailed comparative analysis. (Ba-b) Sections of a ganglion are showing ac-tubulin staining in neuropil (white arrowhead) and commissures (open arrowhead). (Bc) The graph showing Corrected Total Cell Fluorescence (CTCF) levels of section view (0.1% DMSO & 10 uM DAPT). (C) RT-PCR and mRNA levels of Notch transcription factors *Hau-hes* and *Hau-hey* before and after treatment with 10 µM DAPT. Statistical significance was determined by performing a Student's *t*-test; **P < 0.01; ***P < 0.001. Scale bar: 200 μm (A: lateral view); 50 μm (A: ventral view); and 25 μm (B: section view).

We have found that Notch components are expressed in the developing nerve system during organogenesis. For the functional approach of the Notch pathway, how they contribute to nerve system development, we treated the embryos with DAPT, which targets γ-secretase and induces an inhibitory phenotype of the Notch pathway (Gazave et al. 2017). In the negative control, developing anterior (AA), mediator (MA), and posterior (PP) nerve branches are clearly visible in each segmental ganglion (Shain et al. 2004). In 1 μM DAPT, we couldn’t find significant changes in neuron subsets. However, 10 μM concentration of DAPT starts to show a deficiency of axon growth with connective neurons in each segmental ganglion and abnormal nerve fiber distribution in the developing proboscis. In 25 μM concentration, the sample showed high mortality, but we found phenotype showing disturbance of ventral nerve cord (VNC) formation and axon growth (Figure 4A). Along with the immunostaining result, we hypothesized based on the neuron expression in the lateral packet that DAPT treatment can give a clear difference in cell differentiation in VNC. Thus, we measured identifiable expression of acetylated-tubulin in the M8 segment (Figure 4Ba, 4Bb). In cross-section view, we found degeneration of tubulin expression in presumptive neuropil and lateral packets, and the fluorescence level is significantly decreased (Figure 4Bc). To evaluate the difference between two (0.1% DMSO, 10⁠ μM DAPT) expression levels in detail, CTCF (corrected total cell fluorescence) was carried out. We measured the intensity of fluorescence in section view by using ImageJ (a Java-based image analysis software developed at the National Institutes of Health). It was confirmed that the fluorescence intensity of 0.1% DMSO was 7.5 +/ – 0.4 (mean +/ – s.d.; Figure 4Ba), while 10⁠ μM DAPT decreased to 2.66 +/ – 0.39 (Figure 4Bb; *p* < 0.001 by using Welch’s t-test). Additionally, to validate the effects of DAPT drug treatment, mRNA expression levels of *Hau-hes* and *Hau-hey* were identified through qPCR (Figure 4C). Interestingly, the mRNA expression levels for *Hau-hes* and *Hau-hey* were more than 60% decreased after treatment with DAPT (*Hau-hes*; *p* < 0.01, *Hau-hey*; *p* < 0.001 by using Student’s *t*-test)

## Discussion

The Notch signaling pathway has been widely studied in various model organisms, including *Drosophila melanogaster, Caenorhabditis elegans*, and vertebrates (Baonza and Garcia-Bellido 2000; Louvi and Artavanis-Tsakonas 2006; He et al. 2021). However, studies investigating this pathway in lophotrochozoans remain scarce. To enhance our understanding of Notch signaling in this clade, we identified and characterized core Notch signaling components in the Glossiphoniid leech *H. austinensis*. Furthermore, phylogenetic analysis confirmed the evolutionary conservation of these components within lophotrochozoans, indicating that the fundamental elements of the Notch signaling pathway are conserved across metazoans (Figure 1B, S1).

One of the most significant roles of the Notch signaling pathway in metazoans is its involvement in somitogenesis and neural development (Wahi et al. 2016; Engler et al. 2018). In this study, we examined the expression patterns of key Notch components, including the receptor (*Hau-notch1*), ligands (*Hau-delta* and *Hau-jagged*), and transcription factors (*Hau-hes* and *Hau-hey*), from embryonic stage 8 to stage 11. Notably, our findings revealed the dynamic expression patterns of all Notch components during the neural development of *H. austinensis*. During leech embryogenesis, stage 8 is marked by the formation of the germinal plate, which signifies the onset of the segmented body plan of the leech (Kuo and Weisblat 2011; Weisblat and Kuo 2014). The germinal plate serves as the foundational structure for body patterning, laying the groundwork for subsequent tissue differentiation and organogenesis. As the embryo progresses from early to mid-stage 8, the germinal bands coalesce in a zipper-like manner along the future ventral midline in an anteroposterior (A-P) direction, ultimately forming the germinal plate (Kwak et al. 2023). The expression of Notch components along the germinal plate during early- and mid-stage 8 suggests that Notch signaling may play a role in germinal plate formation and the A-P progression of the embryo (Figure 2). Furthermore, their expression in the anterior clusters of cells, which serve as segmental precursors, indicates a potential role for Notch signaling in somitogenesis during the epiboly stage. Also the anterior cells of germinal band differentiate to non-segmental cephalic mesoderm and prostomium, which is related to segmental nervous system (Weisblat and Kuo 2014).

To further investigate the role of Notch components in the neural development of *H. austinensis*, we examined their expression patterns from stages 9 to 11. By stage 9, the A-P axis of the embryo is fully established (Cho et al. 2010; Kuo and Weisblat 2011). At this stage, Notch components were expressed along the somites in the anterior part of the embryo, as well as in the dorsal ganglia, ventral segmental ganglia, and micromere-derived supraesophageal ganglia within the prostomium (Figure 3). These expression patterns strongly suggest the involvement of Notch signaling in neural development. As development progresses to stages 10 and 11, the expression of Notch components becomes evident in the anterior and posterior suckers, proboscis, dorsal midline, and ventral segmental ganglia. These findings further support the role of Notch signaling in neural development and somitogenesis in leeches. Interestingly, in addition to the Notch core components, our study also revealed spatiotemporal expression patterns of the transcription factors *Hau-hes* and *Hau-hey* during leech embryogenesis. *hes* and *Hey* function as transcriptional repressors and play crucial roles in various biological processes, including cell differentiation, proliferation, and apoptosis (Hu and Zou 2022). In Drosophila, *Hes* and *Hey* are involved in segmentation and neural development (Monastirioti et al. 2010, 2024). The expression of *Hau-hes* and *Hau-hey* in the anterior and posterior suckers, as well as in the ventral segmental ganglia, suggests a possible functional similarity to these previous findings.

To further investigate the involvement of Notch pathway components in neural development in leeches, we treated *H. austinensis* embryos at stage 7 with the γ-secretase inhibitor DAPT (Dorneburg et al. 2016; Zhang et al. 2018). DAPT treatment resulted in a marked reduction of α-tubulin staining, indicating disrupted formation of the neuropil and commissures, as well as impaired development of the VNC. Similar inhibitory effects of DAPT on Notch signaling have been observed in planarians (Dong et al. 2021). Consistently, previous studies have shown that DAPT suppresses the expression of Notch, Hes1, and Hes5 (Zhang et al. 2018). These findings suggest that tightly regulated Notch signaling is essential for proper neurogenesis. Excessive inhibition, as seen with DAPT treatment, may lead to aberrant tissue patterning and neurodevelopmental defects during leech embryogenesis (Figure 4).

Overall, this study highlights the crucial role of the Notch signaling pathway in somitogenesis and neural development during leech embryogenesis. The phylogenetic analysis provides insights into the evolutionary relationships of Notch components across metazoans. By characterizing the spatiotemporal expression patterns of Notch signaling molecules throughout various stages of leech development, our findings suggest that Notch signaling plays a fundamental role in neurogenesis and segment formation within lophotrochozoans. These findings contribute valuable evidence to the broader field of evolutionary developmental biology (evo-devo), particularly in understanding the conserved mechanisms of neurogenesis and segmentation within Bilateria.

## Supplementary Material

Supplementary_Table_1.docx

Supplementary_Material_Figure_2.jpg

Supplementary_Material_Figure_1.jpg

## Data Availability

All data generated for the present study are included in the manuscript. The datasets used during the current study are available from the corresponding author upon reasonable request.
