# Supplementary material for: Notch signaling in leech neurogenesis: expression patterns and functional insights in the Glossiphoniid leech Helobdella austinensis
Source: Anim Cells Syst (Seoul). 2026 Feb 23;30(1):282–94. doi: 10.1080/19768354.2026.2631845 (PMC12983829; doi:10.1080/19768354.2026.2631845)
Supplement: Supplementary_Table_1.docx [file TACS_A_2631845_SM3407.docx]

| **Gene** | **Accession number** | **Primer sequence** | | **TM(℃)** | **Remarks** |
| --- | --- | --- | --- | --- | --- |
| *Hau-notch1* | PV360691 | Forward | 5’-CGAGGAGGCTGTTGATTGGT-3’ | 62.7℃ | Probe  synthesis |
|  |  | Reverse | 5’-CAAGCAGGTGTCTTTGTGGC-3’ | 61.5℃ |  |
| *Hau-notch2* | PX097358 | Forward | 5’-CCATTGCCCGAAAAACAGCA-3’ | 61.3℃ | Probe  synthesis |
|  |  | Reverse | 5’-TTTCATCCGTGCCTGAACGA-3’ | 61.7℃ |  |
| *Hau-jagged* | PV360692 | Forward | 5’-AGGGAGTTGCGTTCTCAAGG-3’ | 62.8℃ | Probe  synthesis |
|  |  | Reverse | 5’-AGTAGTTCTGGCACGGGTTG-3’ | 62.1℃ |  |
| *Hau-delta* | PV360694 | Forward | 5’-AGCCGTGTCTTAATGGAGGC-3’ | 61.9℃ | Probe  synthesis |
|  |  | Reverse | 5’-CGTTAAAGCACGGATGGCTG-3’ | 60.9℃ |  |
| *Hau-hes* | PV360695 | Forward | 5’-GAGGGAGTTGAAGGTGCTCG-3’ | 63.9℃ | Probe  synthesis |
|  |  | Reverse | 5’-ACCACCCATATTCCAGCGTG-3’ | 62.5℃ |  |
|  |  | Forward | 5’-ATCAACACCATCGGAGAATGG-3’ | 60.0℃ | qPCR |
|  |  | Reverse | 5’-GGGTGGTCACTCTAATCATCG-3’ | 60.4℃ |  |
| *Hau-hey* | PV360693 | Forward | 5’-CACCCACATCCCACACAACT-3’ | 63.4℃ | Probe  synthesis |
|  |  | Reverse | 5’-TGGAGCGTAGGGGTACTGAG-3’ | 64.1℃ |  |
|  |  | Forward | 5’-CTTGCACGATACAACGCCAC-3’ | 60.5℃ | qPCR |
|  |  | Reverse | 5’-CCTCCATCATCGACCACTTC-3’ | 61.2℃ |  |
