# Supplementary figures and images for: Notch signaling in leech neurogenesis: expression patterns and functional insights in the Glossiphoniid leech Helobdella austinensis
Source: Anim Cells Syst (Seoul). 2026 Feb 23;30(1):282–94. doi: 10.1080/19768354.2026.2631845 (PMC12983829; doi:10.1080/19768354.2026.2631845)

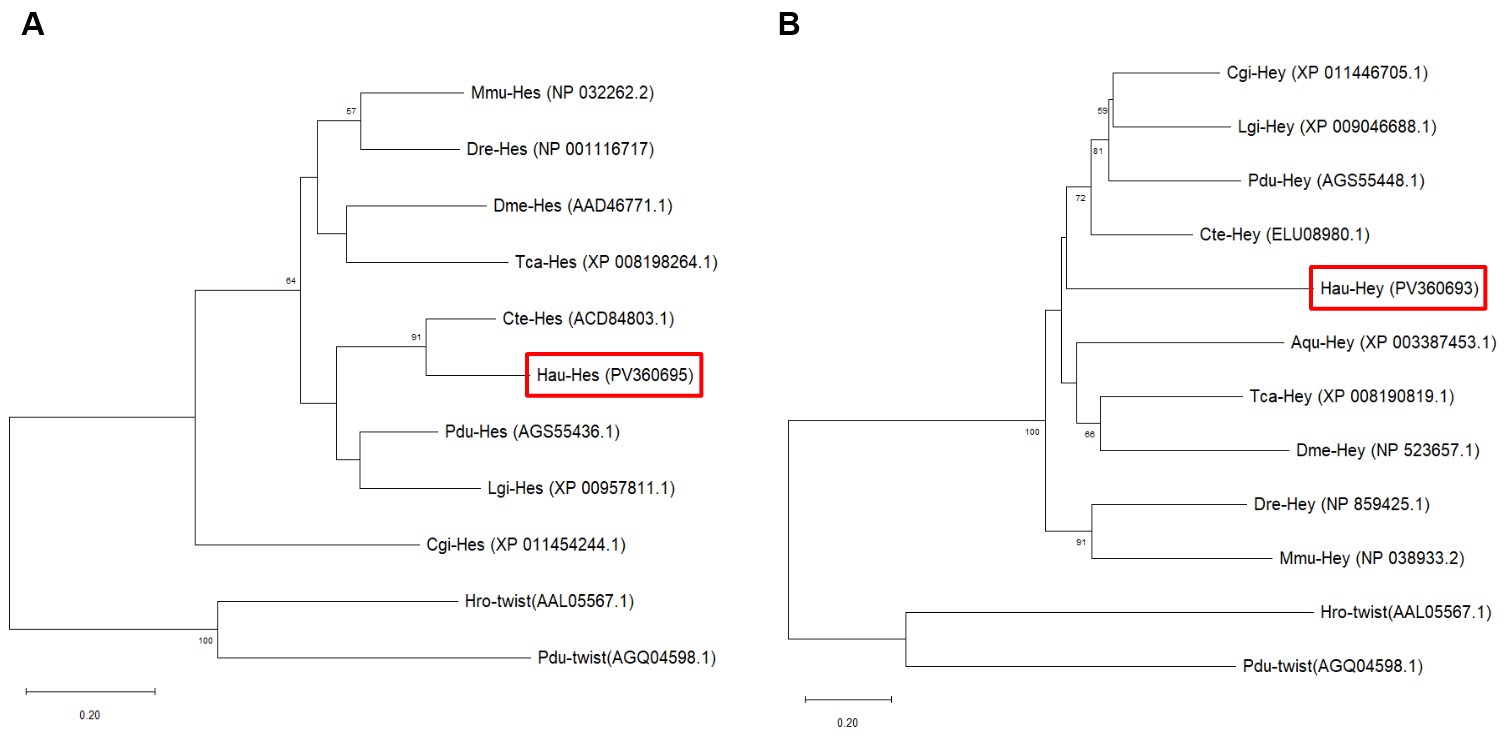

Supplement: Supplementary_Material_Figure_2.jpg [file TACS_A_2631845_SM3406.jpg]

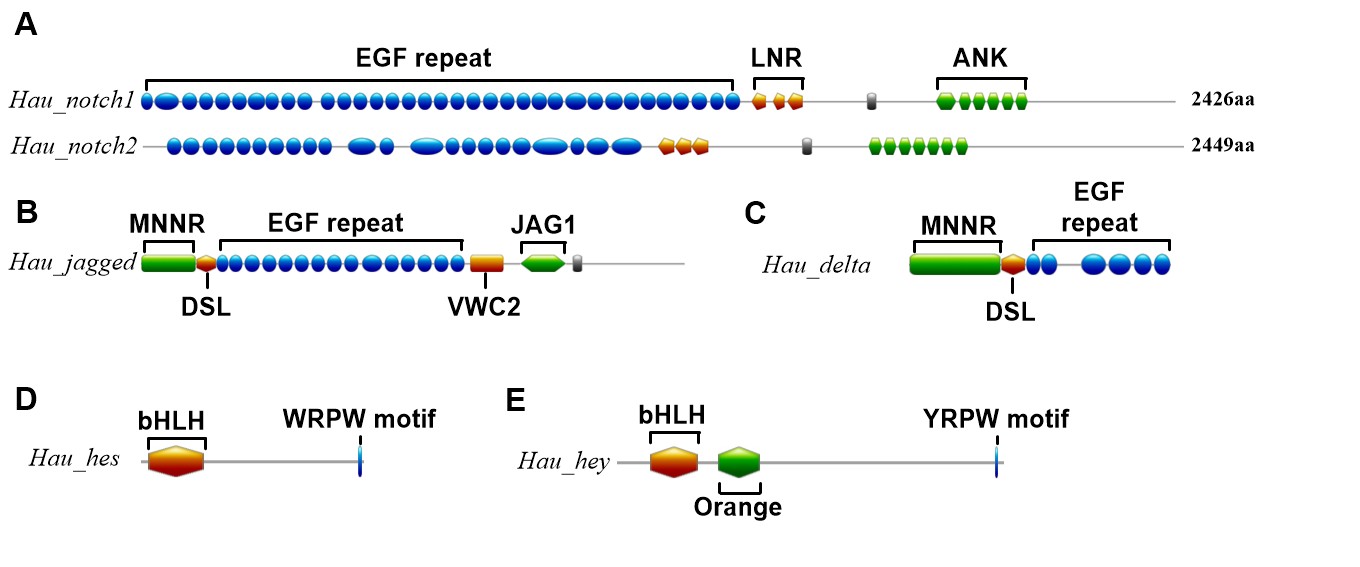

Supplement: Supplementary_Material_Figure_1.jpg [file TACS_A_2631845_SM3405.jpg]
